# Assessment of early and late dysphagia using videofluoroscopy and quality of life questionnaires in patients with head and neck cancer treated with radiation therapy

**DOI:** 10.1186/1748-717X-9-137

**Published:** 2014-06-14

**Authors:** Eda Yirmibeşoğlu Erkal, Doğu Canoğlu, Ahmet Kaya, Görkem Aksu, Binnaz Sarper, Gür Akansel, Tülay Meydancı, Haldun Şükrü Erkal

**Affiliations:** 1 Departments of Radiation Oncology, Kocaeli University Faculty of Medicine, Kocaeli, Turkey; 2 Departments of Radiology, Kocaeli University Faculty of Medicine, Kocaeli, Turkey; 3 Department of Radiation Oncology, Sakarya University Faculty of Medicine, Sakarya, Turkey

**Keywords:** Head and neck cancer, Radiation therapy, Dysphagia, Videofluoroscopy, Quality of life

## Abstract

**Backgorund:**

The aim of this study was to evaluate dysphagia in patients with head and neck cancer (HNC) undergoing three-dimensional conformal radiation therapy using objective and subjective tools simultaneously and to associate the clinical correlates of dysphagia with dosimetric parameters.

**Methods:**

Twenty patients were included in the study. The primary tumor and the involved lymph nodes (LN) were treated with 66-70 Gy, the uninvolved LN were treated with 46-50 Gy. Six swallowing structures were identified: the superior pharyngeal constrictor muscle (SPCM), the middle pharyngeal constrictor muscle (MPCM), the inferior pharyngeal constrictor muscle (IPCM), the base of tongue (BOT), the larynx and the proximal esophageal sphincter (PES). Dysphagia was evaluated using videofluoroscopy and European Organization for Research and Treatment of Cancer (EORTC) QoL questionnaire (QLQ-C30) and supplemental EORTC QoL module for HNC (QLQ-H&N35). The evaluations were performed before treatment, at 3 months and at 6 months following treatment.

**Results:**

On objective evaluation, the D_max_ for the larynx and the sub-structures of the PCM were correlated with impaired lingual movement, BOT weakness and proximal esophageal stricture at 3 months, whereas the V_65_, the V_70_and the D_max_ for the larynx was correlated with BOT weakness and the V_65_, the V_70_, the D_max_ or the D_mean_ for the sub-structures of the PCM were correlated with impaired lingual movement, BOT weakness, reduced laryngeal elevation, reduced epiglottic inversion and aspiration at 6 months following treatment. On subjective evaluation, the V_60_, the D_max_ and the D_mean_ for SPCM were correlated with QoL scores for HNSO at 3 months, whereas the V_70_ for SPCM were correlated with QoL scores for HNPA and the V_60_, the V_65_, the V_70_, the D_max_ and the D_mean_ for SPCM were correlated with QoL scores for HNSO at 6 months following treatment.

**Conclusions:**

The use of multiple dysphagia-related endpoints to complement eachother rather than to overlap with one another, as well as the use of multiple evaluations over time to represent a scale of early to late findings might provide a better insight in terms of the association of the clinical correlates of dysphagia with the dose-volume data for the dysphagia-related anatomical structures.

## Introduction

For head and neck cancer (HNC), the intensification of radiation therapy through the use of altered fractionation schedules and/or concomitant chemotherapy has resulted in significantly improved local control and survival rates. However, these intensified regimens are associated with increased rates of early as well as late dysphagia with presumably prolonged duration [1-3]. Patients with HNC might experience dysphagia either due to their disease or as a consequence of their treatment. Dsyphagia following radiation therapy for HNC might have a significant detrimental effect on the quality-of-life (QoL) [4]. Besides dysphagia, the intensified regimens might be associated with laryngeal edema that promotes aspiration and an increased risk of pneumonia. The recognition of the association between the intensification of the treatment regimens and the worsening of dysphagia has led to the evaluation of the correlations between dysphagia and the dose-volume data for the swallowing-related structures, exploiting the possibility of minimizing the dose delivered to these structures [5-7].

An intrinsic problem associated with such evaluations is the lack of a consensus regarding the timing of dysphagia, since it can be evaluated either as an acute toxicity or as a late toxicity, as well as the definition of dysphagia, since it can be evaluated either objectively through invasive techniques such as videofluoroscopy (VF) and fiberoptic endoscopic evaluation of swallowing (FEES) or subjectively through several specific validated QoL questionnaires, as a treatment related toxicity end point [3]. Hence, the aim of this study was to evaluate both early and late dysphagia in patients with HNC undergoing three-dimensional conformal radiation therapy (3DCRT) using objective and subjective tools simultaneously and to associate the clinical correlates of dysphagia with dosimetric parameters.

## Materials and method

The study design was approved by the Institutional Review Board of Kocaeli University and all of the patients were required to provide written informed consent regarding their participation in the study. Twenty patients with HNC who would receive definitive treatment with 3DCRT with or without concomitant chemotherapy were included in the study. There were 17 males and three females. Their ages ranged from 30 to 76 years (median, 63 years). Tumor localization was the nasopharynx in 10 patients and the supraglottic larynx in 10 patients. Twelve patients received concomitant chemotherapy whereas eight patients did not receive concomitant chemotherapy.

### Treatment

The patient was placed in the supine position and the head was immobilized with a customized thermoplastic mask. The neck was extended and the shoulders were relaxed downward with gentle traction. For radiation therapy planning purposes, computed tomography (CT) images from the vertex to below the clavicles were obtained with a 16-slice scanner using a slice thickness of 3 mm. Intravenous contrast was not used. Radiation therapy was delivered through parallel-opposed lateral portals encompassing the primary tumor and the cervical lymph nodes, matched with an anterior portal encompassing the supraclavicular lymph nodes. The primary tumor was treated with 70 Gy, the involved cervical lymph nodes were treated with 66 to 70 Gy (median, 70 Gy), the uninvolved cervical lymph nodes were treated with 50 to 60 Gy (median, 60 Gy) and the supraclavicular lymph nodes were treated with 46 to 50 Gy (median, 50 Gy) using daily fractions of 2 Gy.

### Evaluation of dose-volume data

The planning CT images and the 3DCRT plans were retrieved from the treatment planning system (XiO, CMS Inc., St. Louis, MO, USA) for the retrospective delineation of the swallowing structures at risk and the respective dose versus volume analysis using dose-volume histograms (DVH). Based on a literature search, six swallowing structures were identified: the superior pharyngeal constrictor muscle (SPCM), the middle pharyngeal constrictor muscle (MPCM), the inferior pharyngeal constrictor muscle (IPCM), the base of tongue (BOT), the larynx and the proximal esophageal sphincter (PES). The mean dose (D_mean_) and the maximum dose (D_max_) to each of these structures and the partial volumes of each of these structures receiving a specified dose (V_D_S) were calculated from the DVHs (Table 1, Table 2 and Table 3). The maximum dose was defined as the dose received by 0.1 cc of the respective structure.

**Table 1:** **The mean dose (D**_**mean**_**) and the maximum dose (D**_**max**_**) to each of the swallowing structures and the partial volumes of each of these structures receiving a specified dose (V**_**D**_**S) as calculated from the dose-volume histograms (DVH) for the entire group of patients**

	**Dose-volume data**				
**Swallowing structure**	**Dmean (Gy) Mean ± SD (range)**	**Dmax (Gy) Mean ± SD (range)**	**V60 (%) Mean ± SD (range)**	**V65 (%) Mean ± SD (range)**	**V70 (%) Mean ± SD (range)**
**PCM**	61.96 ± 7.90 (33.26 - 69.96)	72.61 ± 2.55 (64.88 - 76.79)	74.57 ± 19.83 (31.00 - 99.92)	51.31 ± 24.47 (0.00 - 90.38)	22.24 ± 19.50 (0.00 - 65.33)
**SPCM**	60.66 ± 11.85 (22.94 - 71.65)	70.55 ± 4.56 (58.34 - 76.79)	70.83 ± 34.87 (0.00 - 100.00)	49.13 ± 41.45 (0.00 - 100.00)	22.23 ± 28.19 (0.00 - 83.03)
**MPCM**	66.65 ± 3.51 (61.28 - 74.03)	69.63 ± 3.72 (63.47 - 76.79)	98.23 ± 4.79 (79.56 - 100.00)	61.56 ± 42.23 (0.00 - 100.00)	22.97 ± 37.04 (0.00 - 100.00)
**IPCM**	63.58 ± 6.06 (50.39 - 71.55)	68.61 ± 4.30 (61.27 - 75.99)	75.83 ± 31.97 (2.35 - 100.00)	46.90 ± 45.55 (0.00 - 100.00)	16.51 ± 29.57 (0.00 - 88.40)
**BOT**	50.75 ± 12.52 (23.71 - 71.49)	69.39 ± 4.90 (53.82 - 74.83)	49.38 ± 31.76 (0.00 - 99.99)	27.05 ± 30.75 (0.00 - 99.97)	8.38 ± 18.41 (0.00 - 77.40)
**Larynx**	66.84 ± 6.37 (50.56 - 74.89)	72.64 ± 4.00 (64.45 - 79.30)	87.11 ± 23.21 (29.27 - 100.00)	69.66 ± 33.84 (0.00 - 100.00)	39.25 ± 40.02 (0.00 - 100.00)
**PES**	50.48 ± 9.83 (25.41 - 66.83)	59.63 ± 7.65 (47.11 - 69.85)	17.45 ± 27.58 (0.00 - 100.00)	10.43 ± 23.51 (0.00 - 99.32)	0.00

The pharyngeal constrictor muscle: PCM, the superior pharyngeal constrictor muscle: SPCM, the middle pharyngeal constrictor muscle: MPCM, the inferior pharyngeal constrictor muscle: IPCM, the base of tongue: BOT and the proximal esophageal sphincter: PES.

**Table 2:** **The mean dose (D**_**mean**_**) and the maximum dose (D**_**max**_**) to each of the swallowing structures and the partial volumes of each of these structures receiving a specified dose (V**_**D**_**S) as calculated from the dose-volume histograms (DVH) for patients with nasopharyngeal primaries**

	**Dose-volume data**				
**Swallowing structure**	**Dmean (Gy) Mean ± SD (range)**	**Dmax (Gy) Mean ± SD (range)**	**V60 (%) Mean ± SD (range)**	**V65 (%) Mean ± SD (range)**	**V70 (%) Mean ± SD (range)**
**PCM**	66.03 ± 2.19 (63.17 - 69.96)	72.69 ± 1.62 (69.92 - 74.85)	89.24 ± 9.20 (73.27 - 99.92)	64.75 ± 22.79 (20.19 - 90.38)	27.91 ± 21.83 (0.00-65.33)
**SPCM**	68.35 ± 2.66 (63.29 - 71.65)	72.66 ± 1.64 (69.92 - 74.85)	99.14 ± 1.64 (94.98 - 100.00)	81.35 ± 26.41 (24.37 - 100.00)	37.66 ± 30.09 (0.00-83.03)
**MPCM**	65.30 ± 2.77 (61.28 - 69.62)	67.69 ± 2.60 (64.23 - 72.03)	97.63 ± 6.43 (79.56 - 100.00)	51.33 ± 45.38 (0.00 - 100.00)	4.80 ± 11.93 (0.00-37.40)
**IPCM**	59.96 ± 4.09 (50.39 - 63.35)	65.86 ± 3.18 (61.27 - 71.83)	58.50 ± 34.43 (2.35 - 100.00)	12.09 ± 19.33 (0.00-48.71)	0.62 ± 1.97 (0.00-6.22)
**BOT**	52.85 ± 13.03 (36.80 - 68.08)	70.02 ± 2.79 (65.36 - 74.00)	62.38 ± 32.78 (15–99.55)	34.12 ± 31.63 (0.01-90.03)	5.54 ± 11.05 (0.00-34.86)
**Larynx**	62.55 ± 5.76 (50.56 - 68.07)	70.43 ± 2.97 (64.45 - 73.54)	74.92 ± 28.33 (29.27 - 100.00)	52.05 ± 31.83 (0.00-89.83)	9.52 ± 12.30 (0.00-32.72)
**PES**	50.97 ± 3.22 (46.09 - 57.15)	53.78 ± 4.76 (47.11 - 61.75)	2.08 ± 4.40 (0.00 - 11.10)	0.00	0.00

The pharyngeal constrictor muscle: PCM, the superior pharyngeal constrictor muscle: SPCM, the middle pharyngeal constrictor muscle: MPCM, the inferior pharyngeal constrictor muscle: IPCM, the base of tongue: BOT and the proximal esophageal sphincter: PES.

**Table 3:** **The mean dose (D**_**mean**_**) and the maximum dose (D**_**max**_**) to each of the swallowing structures and the partial volumes of each of these structures receiving a specified dose (V**_**D**_**S) as calculated from the dose-volume histograms (DVH) for patients with laryngeal primaries**

	**Dose-volume data**				
**Swallowing structure**	**Dmean (Gy) Mean ± SD (range)**	**Dmax (Gy) Mean ± SD (range)**	**V60 (%) Mean ± SD (range)**	**V65 (%) Mean ± SD (range)**	**V70 (%) Mean ± SD (range)**
**PCM**	57.89 ± 9.49 (33.26 - 67.07)	72.53 ± 3.33 (64.88 – 76.79)	59.91 ± 16.35 (31.00 – 82.97)	37.87 ± 18.54 (0.00 – 70.75)	16.58 ± 15.97 (0.00 – 40.19)
**SPCM**	52.97 ± 12.57 (22.94 – 68.71)	68.46 ± 5.61 (58.34 – 76.79)	42.52 ± 27.99 (0.00 – 90.38)	16.92 ± 24.95 (0.00 – 90.38)	16.92 ± 24.95 (0.00 – 84.11)
**MPCM**	67.70 ± 3.78 (62.41 – 74.03)	71.58 ± 3.74 (63.47 – 76.79)	98.84 ± 2.51 (93.06 – 100.00)	71.79 ± 38.37 (0.00 – 100.00)	41.15 ± 44.95 (0.00 – 100.00)
**IPCM**	68.20 ± 3.64 (61.52 – 71.55)	71.35 ± 3.48 (63.32 – 75.99)	93.15 ± 17.48 (44.84 – 100.00)	81.71 ± 36.25 (0.00 – 100.00)	32.39 ± 35.80 (0.00 – 88.40)
**BOT**	48.65 ± 12.29 (23.71 – 71.49)	68.75 ± 6.49 (53.82 – 74.83)	36.37 ± 26.09 (0.00 – 99.99)	19.99 ± 29.75 (0.00 – 99.97)	11.21 ± 23.99 (0.00 – 77.40)
**Larynx**	71.13 ± 3.34 (64.16 – 74.89)	74.85 ± 3.75 (66.42 – 79.30)	99.30 ± 2.03 (93.54 – 100.00)	87.27 ± 26.73 (20.00 – 100.00)	68.98 ± 35.57 (0.00 – 100.00)
**PES**	49.99 ± 13.89 (25.41 – 66.83)	65.48 ± 4.97 (55.77 – 69.85)	32.83 ± 32.58 (0.00 – 100.00)	20.86 ± 30.41 (0.00 – 99.32)	0.00

The pharyngeal constrictor muscle: PCM, the superior pharyngeal constrictor muscle: SPCM, the middle pharyngeal constrictor muscle: MPCM, the inferior pharyngeal constrictor muscle: IPCM, the base of tongue: BOT and the proximal esophageal sphincter: PES.

### Evaluation of dysphagia

Dysphagia was evaluated objectively using VF and subjectively using the European Organization for Research and Treatment of Cancer (EORTC) QoL questionnaire (QLQ-C30) and the supplemental EORTC QoL module for HNC (QLQ-H&N35). The evaluations were performed before treatment, at 3 months following treatment and at 6 months following treatment.

### Videofluoroscopy

For VF, the patient was seated and viewed in the lateral plane and the anteroposterior plane for images of the lips anteriorly, the cervical vertebra posteriorly, the soft palate superiorly and the lower end of the cervical esophagus inferiorly. The patient was asked to swallow thin liquid barium (diluted with water) followed with non-diluted barium. A timer was activated at the start of the examination and the examination was monitored and recorded for the evaluation to be completed subsequently. The examination was interpreted separately by two radiologists regarding the manipulation, the control and the passage of the bolus, with the focus on cohesion, motility and timing.

Impaired lingual movement, BOT weakness, pharyngeal residue, reduced laryngeal elevation, reduced epiglottic inversion, swallow reflex delay, cricopharyngeal muscle (CM) dysfunction, proximal esophageal stricture, penetration and aspiration were evaluated under VF (Table 4). Impaired lingual movement was defined as rolling movements of the tongue with repetitive attempts at initiating swallowing, resulting in some portion of the bolus remaining in the mouth. BOT weakness was defined as the loss of contact of the base of tongue with the posterior pharyngeal wall due to reduced motion and the resultant worsening of the propulsion of the bolus. Pharyngeal residue was defined as any portion of the bolus remaining in the vallecula or the pyriform sinuses after the swallow, with the potential risk of aspiration after the swallow. Reduced laryngeal elevation was determined by measuring the extent of maximal laryngeal rise during swallow. Reduced epiglottic inversion was defined as the diminished degree of movement of the epiglottis from vertical to horizontal during swallowing. Swallow reflex delay was defined as the duration of the swallowing phase beyond the range found in normal controls. CM dysfunction was defined as the indentation of the CM into the lumen of the hypopharynx, incomplete relaxation and premature closure. Proximal esophageal stricture was defined based on the size of the narrowest point of opening of the PES during maximal distention in the proximal esophagus for bolus passage. Penetration was defined as any portion of the bolus entering the laryngeal vestibule to the level of (but not passing below) the vocal folds. Aspiration was defined as occurring once the bolus passed the level of the vocal folds and entered the subglottic region.

**Table 4:** The videofluoroscopy findings regarding impaired lingual movement, base of tongue (BOT) weakness, pharyngeal residue, reduced laryngeal elevation, reduced epiglottic inversion, swallow reflex delay, cricopharyngeal muscle (CM) dysfunction, proximal esophageal stricture, penetration and aspiration before treatment, at 3 months following treatment and at 6 months following treatment

	**Evaluation period**		
**Videofluoroscopy finding**	**Before treatment n (%)**	**At 3 months following treatment n (%)**	**At 6 months following treatment n (%)**
**Impaired lingual movement**	1 (5)	4 (20)	2 (10)
**BOT weakness**	10 (50)	15 (75)	12 (60)
**Pharyngeal residue**	7 (35)	11 (55)	10 (50)
**Reduced laryngeal elevation**	2 (10)	2 (10)	3 (15)
**Reduced epiglottic inversion**	0 (0)	6 (30)	5 (25)
**Swallow reflex delay**	0 (0)	1 (5)	1 (5)
**CM dysfunction**	0 (0)	2 (10)	2 (10)
**Proximal esophageal stricture**	3 (15)	5 (25)	6 (30)
**Penetration**	5 (25)	10 (50)	14 (70)
**Aspiration**	0 (0)	1 (5)	4 (20)

### Quality-of-life

The patient was asked to independently fill in the Turkish translation of the EORTC QLQ-C30 (Version 3.0) consisting of 30 items and the supplemental EORTC QLQ-H&N35 consisting of 35 items. The EORTC QLQ-C30 [8] was used to evaluate the general QoL using the global health status/QoL (QL) constructed scale, whereas the EORTC QLQ-H&N35 [9] was used to evaluate the dysphagia-related QoL using the pain (HNPA), the swallowing (HNSW) and the trouble with social eating (HNSO) symptom scales (Table 5). Regarding the EORTC QLQ-C30, the higher scores represented better responses in terms of the general QoL, whereas regarding the EORTC QLQ-H&N35, the lower scores represented better responses in terms of the dysphagia-related QoL.

**Table 5:** The quality-of-life (QoL) scores regarding the general QoL using the global health status/QoL (QL) constructed scale and the dysphagia-related QoL using the pain symptom scale (HNPA), the swallowing symptom scale (HNSW) and the trouble with social eating symptom scale (HNSO) at 3 months following treatment and at 6 months following treatment

	**Evaluation period**		
**QoL score**	**Before treatment Mean ± SD (range)**	**At 3 months following treatment Mean ± SD (range)**	**At 6 months following treatment Mean ± SD (range)**
**QL**	65 ± 24 (17–20)	67 ± 15 (33–83)	66 ± 19 (17–92)
**HNPA**	18 ± 24 (0–75)	17 ± 28 (0–100)	12 ± 19 (0–58)
**HNSW**	21 ± 20 (0–58)	36 ± 21 (0–100)	34 ± 24 (0–100)
**HNSO**	21 ± 22 (0–75)	33 ± 28 (0–100)	33 ± 30 (0–92)

### Statistical analysis

Statistical analysis was performed using SPSS for Windows version 17.0 (SPSS Inc., 2008, Chicago, USA). McNemar’s test was used to compare VF findings and paired samples *t*-test was used to compare QoL scores. Mann–Whitney *U* test was used to correlate dose-volume data and QoL scores with VF findings. Pearson product–moment correlation was used to correlate dose-volume data with QoL scores. Statistical significance was considered when the p-value was 0.05 and below.

## Results

### Objective evaluation of dysphagia using VF

The difference in terms of the VF finding of reduced epiglottic inversion before treatment as compared to that finding at 3 months following treatment was statistically significant (p = 0.03). The difference in terms of the VF finding of penetration before treatment as compared to that finding at 6 months following treatment was statistically significant (p = 0.004). There was no statistically significant difference when the patients were grouped according their tumor localization (the nasopharynx versus the supraglottic larynx) or the chemotherapy status (receiving concomitant chemotherapy versus not receiving concomitant chemotherapy).

### Subjective evaluation of dysphagia using QLQ-C30 and QLQ-H&N35

The difference in terms of the HNSW scores before treatment as compared to those scores at 3 months following treatment was statistically significant (p = 0.05). There was no statistically significant difference when the patients were grouped according their tumor localization or the chemotherapy status.

### Dose-volume data correlated with VF findings

When compared according the presence of impaired lingual movement on VF, the difference in terms of D_max_ (69.66 vs 74.15 Gy, p = 0.05) for SPCM at 3 months following treatment and the difference in terms of V_70_ (15.88 vs 86.78%, p = 0.04) for MPCM at 6 months following treatment were statistically significant. When compared according the presence of BOT weakness on VF, the difference in terms of D_max_ (69.24 vs 73.77 Gy, p = 0.03) at 3 months following treatment and in terms of V_65_ (54.43 vs 79.82%, p = 0.03), V_70_ (12.82 vs 56.87%, p = 0.02) and D_max_ (70.26 vs 74.22 Gy, p = 0.03) at 6 months following treatment for the larynx, the difference in terms of V_70_ (11.66 vs 30.51%, p = 0.04), D_mean_ (64.65 vs 67.98 Gy, p = 0.03) and D_max_ (67.49 vs 71.06 Gy, p = 0.03) for MPCM at 6 months following treatment and the difference in terms of V_65_ (15.53 vs 67.81%, p = 0.005), D_mean_ (59.97 vs 65.99 Gy, p = 0.03) and D_max_ (65.76 vs 70.51 Gy, p = 0.02) for IPCM at 6 months following treatment were statistically significant. When compared according the presence of reduced laryngeal elevation on VF, the difference in terms of V_65_ (41.06 vs 94.85%, p = 0.05) at 6 months following treatment for SPCM and the difference in terms of V_65_ (46.31 vs 79.64%, p = 0.04) at 6 months following treatment for PCM were statistically significant. When compared according the presence of reduced epiglottic inversion on VF, the difference in terms of V_70_ (12.48 vs 51.47%, p = 0.01) and D_max_ (69.53 vs 73.62 Gy, p = 0.05) at 6 months following treatment for SPCM and the difference in terms of V_70_ (15.84 vs 41.45%, p = 0.01) and D_mean_ (60.85 vs 65.30 Gy, p = 0.05) at 6 months following treatment for PCM were statistically significant. When compared according the presence of proximal esophageal stricture on VF, the difference in terms of V_70_ (8.74 vs 39.79%, p = 0.04) and D_max_ (67.59 vs 71.66 Gy, p = 0.05) at 3 months following treatment for IPCM were statistically significant. When compared according the presence of aspiration on VF, the difference in terms of V_70_ (14.09 vs 58.51%, p = 0.05) and D_mean_ (65.80 vs 70.04 Gy, p = 0.05) at 6 months following treatment for MPCM were statistically significant.

For patients with nasopharyngeal primaries, when compared according the presence of impaired lingual movement on VF, the difference in terms of D_mean_ (61.26 vs 67.74 Gy, p = 0.04) for larynx at 3 months following treatment was statistically significant. When compared according the presence of BOT weakness on VF, the difference in terms of D_max_ (65.64 vs 68.56 Gy, p = 0.05) for MPCM, D_max_ (62.92 vs 67.12 Gy, p = 0.03) for IPCM, V_70_ (0 vs 13.6%, p = 0.02) and D_max_ (66.77 vs 72.00 Gy, p = 0.02) for larynx at 3 months and interms of D_mean_ (2.93 vs 66.31 Gy, p = 0.03) and D_max_ (65.64 vs 68.56 Gy, p = 0.02) for MPCM, D_max_ (62.92 vs 67.12 Gy, p = 0.01) for IPCM, V_70_ (0 vs 13.6 Gy, p = 0.01) and D_max_ (66.77 vs 72.00 Gy, p = 0.01) for larynx at 6 months following treatment were statistically significant. When compared according the presence of reduced laryngeal elevation on VF, the difference in terms of V_70_ (4.41 vs 29.96%, p = 0.03) for larynx at 3 months and in terms of V_70_ (0 vs 15.99%, p = 0.02) and D_max_ (66.61 vs 70.18 Gy, p = 0.05) for MPCM, D_max_ (64.45 vs 69.15 Gy, p = 0.03) for IPCM, V_70_ (3.31 vs 24.00%, p = 0.03) and D_max_ (69.36 vs 72.93 Gy, p = 0.02) for larynx at 6 months following treatment were statistically significant. When compared according the presence of reduced epiglottic inversion on VF, the difference in terms of V_70_ (14.93 vs 47.38%, p = 0.02) and D_mean_ (64.75 vs 67.96 Gy, p = 0.02) for PCM, V_70_ (20.90 vs 62.80%, p = 0.05) for SPCM at 6 months following treatment were statistically significant. When compared according the presence of proximal esophageal stricture on VF, V_70_ (0 vs 3.11%, p = 0.05) for IPCM and D_max_ (69.71 vs 73.32 Gy, p = 0.04) for larynx at 3 months following treatment and in terms of D_max_ (64.69 vs 68.58Gy, p = 0.05) for IPCM and D_max_ (69.36 vs 72.93 Gy, p = 0.03) for larynx at 6 months following treatment were statistically significant. When compared according the presence of aspiration on VF, D_mean_ (65.98 vs 66.26%, p = 0.04) for PCM, V_70_ (0 vs 3.11%, p = 0.05) for IPCM and D_max_ (69.71 vs 73.32 Gy, p = 0.05) for larynx at 6 months following treatment were statistically significant.

For patients with laryngeal primaries, when compared according the presence of impaired lingual movement on VF, the difference in terms of V_70_ (6.21 vs 9.14%, p = 0.05) and D_max_ (66.88 vs 74.78 Gy, p = 0.05) for SPCM, V_70_ (26.85 vs 98.32%, p = 0.03), D_mean_ (66.79 vs 72.83 Gy, p = 0.04) and D_max_ (70.58 vs 75.56 Gy, p = 0.04) for MPCM at 3 months following treatment were statistically significant. When compared according the presence of proximal esophageal stricture on VF, the difference in terms of D_mean_ (67.09 vs 70.78 Gy, p = 0.05) for IPCM at 3 months and interms of D_mean_ (67.09 vs 70.78 Gy, p = 0.05) for IPCM at 6 months following treatment were statistically significant. When compared according the presence of penetration on VF, the difference in terms of D_max_ (64.44 vs 71.14 Gy, p = 0.05) for SPCM at 6 months following treatment were statistically significant. When compared according the presence of aspiration on VF, the difference in terms of V_70_ (6.21 vs 9.14%, p = 0.05) and D_max_ (66.88 vs 74.78 Gy, p = 0.05) for SPCM at 6 months following treatment were statistically significant.

For patients receiving chemotherapy, when compared according the presence of BOT weakness on VF, the difference in terms of V_60_ (92.10 vs 99.23%, p = 0.05), V_65_ (18.80 vs 70.27%, p = 0.05), V_70_ (0 vs 21.40%, p = 0.05), D_mean_ (62.93 vs 67.50 Gy, p = 0.05) and D_max_ (65.64 vs 71.30 Gy, p = 0.01) for MPCM, V_65_ (0 vs 67.47%, p = 0.01), V_70_ (0 vs 32.82%, p = 0.05), D_mean_ (56.32 vs 65.80 Gy, p = 0.05) and D_max_ (62.92 vs 71.27 Gy, p = 0.01) for IPCM, V_65_ (31.74 vs 80.64%, p = 0.05), V_70_ (0 vs 54.34%, p = 0.01) and D_max_ (66.77 vs 74.44 Gy, p = 0.01) for larynx at 3 months and interms of V_65_ (20.12 vs 84.03%, p = 0.01), V_70_ (0 vs 27.52%, p = 0.009), D_mean_ (63.42 vs 68.46 Gy, p = 0.007) and D_max_ (66.46 vs 72.33 Gy, p = 0.004) for MPCM, D_max_ (65.23 vs 72.00 Gy, p = 0.03) for IPCM, V_70_ (11.74 vs 61.47%, p = 0.02) and D_max_ (69.17 vs 74.91 Gy, p = 0.02) for larynx at 6 months following treatment were statistically significant. When compared according the presence of reduced epiglottic inversion on VF, the difference in terms of V_70_ (20.38 vs 51.54%, p = 0.01) and D_mean_ (63.01 vs 68.18 Gy, p = 0.02) for PCM, V_70_ (14.06 vs 69.14%, p = 0.03) and D_mean_ (61.38 vs 70.55 Gy, p = 0.03) for SPCM at 6 months following treatment were statistically significant. When compared according the presence of proximal esophageal stricture on VF, the difference in terms of V_70_ (1.86 vs 44.44%, p = 0.007), D_mean_ (64.77 vs 69.55 Gy, p = 0.03) and D_max_ (68.18 vs 73.29 Gy, p = 0.02) for MPCM, V_70_ (12.05 vs 49.74%, p = 0.03) and D_max_ (67.23 vs 73.08 Gy, p = 0.05) for IPCM and D_max_ (70.95 vs 75.65 Gy, p = 0.04) for larynx at 3 months following treatment and in terms of V_65_ (32.90 vs 91.70%, p = 0.02), V_70_ (2.12 vs 35.55%, p = 0.04), D_mean_ (64.34 vs 69.18 Gy, p = 0.02) and D_max_ (67.97 vs 72.56 Gy, p = 0.03) for MPCM at 6 months following treatment were statistically significant.

For patients not receiving chemotherapy, when compared according the presence of impaired lingual movement on VF, the difference in terms of V_70_ (15.54 vs 86.78%, p = 0.06) for MPCM, V_70_ (0.33 vs 16.39%, p = 0.02) and D_mean_ (61.94 vs 69.37 Gy, p = 0.05) for IPCM and V_70_ (17.00 vs 97.00%, p = 0.05), D_mean_ (66.29 vs 73.52 Gy, p = 0.05) and D_max_ (71.24 vs 77.56 Gy, p = 0.05) for larynx at 6 months following treatment were statistically significant. When compared according the presence of BOT weakness on VF, the difference in terms of D_mean_ (59.93 vs 66.12 Gy, p = 0.05) for IPCM and D_max_ (53.32 vs 63.58 Gy, p = 0.03) for PES at 6 months following treatment were statistically significant.

### Dose-volume data correlated with QoL scores

At 3 months following treatment, QoL scores for HNSO were positively correlated with V_60_ (p = 0.05, *r* = 0.44), D_mean_ (p = 0.03, *r* = 0.48) and D_max_ for SPCM (p = 0.01, *r* = 0.54). At 6 months following treatment, QoL scores for HNPA were positively correlated with V_70_ for SPCM (p = 0.02, *r* = 0.51) and V_65_ (p = 0.04, *r* = 0.46) and V_70_ (p = 0.03, *r* = 0.50) for PCM and QoL scores for HNSO were positively correlated with V_60_ (p = 0.01, *r* = 0.56), V_65_ (p = 0.002, *r* = 0.65), V_70_ (p = 0.005, *r* = 0.60), D_mean_ (p = 0.008, *r* = 0.57) and D_max_ for SPCM (p = 0.002, *r* = 0.65) and V_60_ (p = 0.02, *r* = 0.50), V_65_ (p = 0.01, *r* = 0.55), V_70_ (p = 0.03, *r* = 0.50) and D_mean_ for PCM (p = 0.02, *r* = 0.51).

For patients with nasopharyngeal primaries, at 3 months following treatment, QoL scores for HNPA were positively correlated with V_70_ (p = 0.03, *r* = 0.69) and D_mean_ (p = 0.005, *r* = 0.81) for PCM and QoL scores for HNSW were positively correlated with D_mean_ (p = 0.03, *r* = 0.68) for BOT. At 6 months following treatment, QoL scores for QL were negatively correlated with D_max_ for PCM (p = 0.04, *r* = −0.66) and D_max_ for SPCM (p = 0.04, *r* = −0.67) and QoL scores for HNPA were positively correlated with V_70_ (p = 0.05, *r* = 0.64) and D_mean_ (p = 0.01, *r* = 0.76) for PCM.

For patients with laryngeal primaries, at 6 months following treatment, QoL scores for HNSO were positively correlated with D_max_ for SPCM (p = 0.04, *r* = 0.65).

For patients receiving chemotherapy, at 3 months following treatment, QoL scores for HNPA were positively correlated with V_70_ (p = 0.03, *r* = 0.62) and D_mean_ (p = 0.04, *r* = 0.60) for PCM and QoL scores for HNSO were positively correlated with D_max_ for SPCM (p = 0.002, *r* = 0.80). At 6 months following treatment, QoL scores for QL were negatively correlated with D_max_ for PCM (p = 0.03, *r* = −0.62) and D_max_ for BOT (p = 0.009, *r* = −0.72), QoL scores for HNPA were positively correlated with V_70_ (p = 0.01, *r* = 0.70) and D_mean_ (p = 0.03, *r* = 0.62) for PCM and V_70_ (p = 0.03, *r* = 0.62) for SPCM, QoL scores for HNSW were positively correlated with V_70_ (p = 0.01, *r* = 0.69) for PCM, V_70_ (p = 0.03, *r* = 0.64) for SPCM and D_max_ for BOT (p = 0.05, *r* = 0.59) and QoL scores for HNSO were positively correlated with V_70_ (p = 0.02, *r* = 0.66) and D_max_ for PCM (p = 0.05, *r* = 0.56), V_65_ (p = 0.02, *r* = 0.65), V_70_ (p = 0.009, *r* = 0.72) and D_max_ (p = 0.002, *r* = 0.79) for SPCM and D_max_ for BOT (p = 0.004, *r* = 0.76).

### VF findings correlated with QoL scores

When compared according the presence of VF findings, the difference in terms of the QoL scores before treatment, at 3 months following treatment and at 6 months following treatment were not statistically significant.

For patients with laryngeal primaries, when compared according the presence of penetration on VF, the difference in terms QoL scores for QL (68.75 vs 25.00, p = 0.04) before treatment and in terms of QoL scores for HNSO (0 vs 26.5, p = 0.05) at 6 months following treatment were statistically significant. When compared according the presence of aspiration on VF, the difference in terms QoL scores for QL (69.88 vs 46.00, p = 0.05) at 6 months following treatment were statistically significant.

For patients receiving chemotherapy, when compared according the presence of reduced epiglottic inversion on VF, the difference in terms QoL scores for HNSW (25 vs 50.33, p = 0.03) at 6 months following treatment were statistically significant.

For patients not receiving chemotherapy, when compared according the presence of reduced epiglottic inversion on VF, the difference in terms QoL scores for QL (69.33 vs 29.50, p = 0.04) at 6 months following treatment was statistically significant. When compared according the presence of aspiration on VF, the difference in terms QoL scores for QL (69.33 vs 29.50, p = 0.04) at 6 months following treatment was statistically significant.

## Discussion

Strong evidence suggests that the intensification of radiation therapy for HNC should result in improved local control and survival. However, such intensification would not be achievable, even through the use of highly conformal radiation therapy techniques, unless the necessary measures are undertaken in attempt to prevent potentially serious treatment related toxicity, most notably in the form of xerostomia and dysphagia [10]. At present, certain measures to reduce the incidence and severity of xerostomia have been successfully implemented into treatment regimens. However, the same does not apply to dysphagia. Hence, dysphagia appears to be arguably the biggest challenge towards further improving the therapeutic index for definitive radiation therapy for HNC [11].

The prevalence of dysphagia in patients receiving definitive radiation therapy for HNC appears to be quite high. Dysphagia might interfere with the nutritional status (leading to gastrostomy tube dependence or a need for esophageal dilatation), compromise the QoL (leading to anxiety and/or depression) and even result in life-threatening complications such as aspiration-related pneumonia [1,5]. Any successful attempt at the reduction of the incidence and the severity of radiation therapy related dysphagia should rely on the physiology of swallowing as well as the clinical and the dosimetric determinants of dysphagia [12]. The complex process of swallowing includes the conscious effort of food ingestion and the subconscious efforts of bolus preparation in the oral cavity and bolus transportation from the pharynx through the esophagus. Swallowing requires the intricate coordination of numerous muscles of the oral cavity, the pharynx, the larynx and the esophagus. In the oral preparatory phase, the tongue helps mixing the food and moving it towards the teeth while the soft palate creates a seal to prevent premature spillage in to the pharynx. The tongue then contracts from the anterior to the posterior, pushing the bolus into the pharynx. In the pharyngeal phase, the soft palate closes the nasopharynx as the larynx is elevated and closed, the PCMs are contracted and the CM is relaxed. The true cords, the false cords, the epiglottis and the aryepiglottic folds constrict to form a barrier to prevent aspiration. When the larynx is elevated superiorly and anteriorly and the CM is relaxed, the resultant negative pressure in the proximal esophagus helps the movement of the bolus. The laryngeal inlet is closed through the elevation of the larynx as well as the pressure exerted by the BOT and bolus itself. In the esophageal phase, a single primary peristaltic wave and several secondary peristaltic waves help to clear the residue and any gastric reflux [13].

Radiation therapy might result in functional impairments involving the entire time frame describing swallowing, such as reduced oromotor control and delayed or absent reflexes prior to swallowing, reduced laryngeal closure, reduced epiglottic inversion or reduced laryngeal elevation during swallowing and reduced pharyngeal peristalsis, CM dysfunction and structural abnormalities following swallowing [14]. The outcome might be a reduction in the lingual range of motion and impaired posterior movement of the BOT regarding the oral phase and the pharyngeal phase, laryngeal incompetence and resultant aspiration regarding the pharyngeal phase and the esophageal phase or the development of an esophageal stricture regarding the esophageal phase [15,16]. Despite the complexity in defining the most important anatomical structures (such as the BOT, the larynx and the PES) and sub-structures (such as the SPCM, the MPCM and the IPCM) whose damage would be the likely cause of dysphagia and the relative paucity in evaluating the associated dose-volume data, dysphagia in relation to dosimetric parameters has been the subject of recent research by many investigators [17,18].

Although a number of studies that evaluated various dysphagia-related endpoints (such as the objective VF findings or the subjective QoL scores) in relation to the dose-volume data for the dysphagia-related anatomical structures (such as the larynx and the pharynx) were reviewed for the Quantitative Analysis of Normal Tissue Effects in the Clinic (QUANTEC), the data from these studies that was used to produce the recommended dose versus volume limits for the larynx and the pharynx mainly apply to patients treated either with 3DCRT or with intensity-modulated radiation therapy (IMRT) not aimed at sparing the dysphagia-related anatomical structures [19]. As a consequence, drawing strict dose versus volume constraints or volume effect parameters from these studies were hampered by the fact that high doses had already been delivered to these structures. Jensen et al. used EORTC QoL questionnaires as the subjective means and FEES as the objective means to describe swallowing function following 3DCRT and attempted to correlate swallowing function with irradiated volumes. Subjective swallowing complaints were present in 83% of the patients whereas 100% of the patients had abnormal FEES findings, most common of which were residuals, penetration, aspiration and decreased sensitivity. Correlations were observed between subjective and objective measures of swallowing dysfunction and the dosimetric parameters of dysphagia-related anatomical structures. Doses less than 60 Gy to the supraglottic larynx and the PES were associated with a lower risk of aspiration [20]. Levendag et al. reported on the use of multiple QoL instruments to objectively evaluate the long-term effects of radiation therapy on the swallowing apparatus on 81 patients with oropharyngeal cancer treated with 3DCRT or IMRT. The probability of patient-rated swallowing complaints was correlated with the D_mean_ for the SPCM and the D_mean_ for the MPCM. Since a sharp increase (of approximately 18%) was observed in patient-rated swallowing complaints per 10 Gy of dose beyond the D_mean_ of 55 Gy, constraining the dose to be received by the swallowing muscles was proposed as a means of improving the QoL [7]. Feng et al. prospectively evaluated dysphagia in patients with oropharyngeal cancer and nasopharyngeal cancer prior to and at 3 months following IMRT for VF-based objective endpoints, patient-reported subjective endpoints and observer-rated subjective endpoints. The D_mean_ for the PCMs and the D_mean_ for the glottic and the supraglottic larynx were correlated with the objective finding of aspiration on VF, the D_mean_ for the PCMs and the D_mean_ for the esophagus were correlated with the subjective finding of worsened liquid swallowing on patient-reported questionnaires and the D_mean_ for the PCMs was correlated with the subjective finding of worsened solid swallowing on both patient-reported questionnaires and observer-rated toxicity scoring [5]. In the present study, dysphagia was prospectively evaluated in patients with nasopharyngeal cancer and supraglottic laryngeal cancer prior to 3DCRT, at 3 months following 3DCRT and at 6 months following 3DCRTusing bothVF-based objective endpoints and patient-reported subjective endpoints. On objective evaluation, the D_max_ for the larynx and the sub-structures of the PCM were correlated with impaired lingual movement, BOT weakness and proximal esophageal stricture at 3 months following treatment, whereas the V_65_, the V_70_and the D_max_ for the larynx was correlated with BOT weakness and the V_65_, the V_70_, the D_max_ or the D_mean_ for the sub-structures of the PCM were correlated with impaired lingual movement, BOT weakness, reduced laryngeal elevation, reduced epiglottic inversion and aspiration at 6 months following treatment. On subjective evaluation, the V_60_, the D_max_ and the D_mean_ for SPCM were correlated with QoL scores for HNSO at 3 months following treatment, whereas the V_70_ for SPCM were correlated with QoL scores for HNPA and the V_60_, the V_65_, the V_70_, the D_max_ and the D_mean_ for SPCM were correlated with QoL scores for HNSO at 6 months following treatment.

## Conclusion

In most of the studies that attempt to correlate the objective VF findings and the subjective QoL scores, there are discrepancies in terms of the dysphagia-related endpoints in relation to the dose-volume data for the dysphagia-related anatomical structures. A possible cause of the discrepancy might be the variations in terms of the methods used in the assessment of dysphagia, ranging from objective means such as aspiration-related radiological imaging and feeding tube dependency to subjective means such as patient-reported questionnaires or observer-reported scales. Another possible cause of the discrepancy might be the patients’ lack of appreciation of certain functional swallowing abnormalities, despite these abnormalities being clinically relevant, and the shifts observed over time in the patients’ responses regarding the subjective endpoints, representing either a true functional improvement or the patients’ accommodation to their new functional status. Therefore, the use of multiple dysphagia-related endpoints to complement each other rather than to overlap with one another, as well as the use of multiple evaluations over time to represent a scale of early to late findings might provide a better insight in terms of the association of the clinical correlates of dysphagia with the dose-volume data for the dysphagia-related anatomical structures.

## Competing interests

The authors declare that they have no competing interests.

## Authors’ contributions

The idea for research generation: EYE, HSE; Planning the methods: EYE, HSE, GA; Supervision and manuscript preparation: EYE, GA, BS, GA; Supplying financial resources and personel vital GA, BS, GA; Responsibility for conducting experiments, management of patients and collecting data: EYE, DC, AK, TM; Responsibility for presentation of results: EYE, DC, AK, HSE; Responsibility for conducting literature search: EYE, DC, AK, HSE; Writting manuscript EYE, HSE; Critical review before submission EYE, DC, AK, GA, BS, GA, TM and HSE. All authors read and approved the final manuscript.
